# Salivary pH Modulation and Antimicrobial Properties of Oregano-Oil Jelly in Relation to Menstrual and Menopausal Status

**DOI:** 10.3390/nu17152480

**Published:** 2025-07-29

**Authors:** Georgiana Ioana Potra Cicalău, Gabriela Ciavoi, Ioana Scrobota, Ionut Daniel Venter, Madalin Florin Ganea, Marc Cristian Ghitea, Evelin Claudia Ghitea, Maria Flavia Gîtea, Timea Claudia Ghitea, Csaba Nagy, Diana Constanta Pelea, Luciana Dobjanschi, Octavia Gligor, Corina Moisa, Mariana Ganea

**Affiliations:** 1 Dental Medicine Department, Faculty of Medicine and Pharmacy, University of Oradea, 1st December Square 10, 410073 Oradea, Romania; cicalau.georgiana@uoradea.ro (G.I.P.C.); gciavoi@uoradea.ro (G.C.); ioana_scrobota@uoradea.ro (I.S.); 2 Doctoral School of Biomedical Sciences, University of Oradea, 1 Universității Street, 410073 Oradea, Romania; venterionutdoctorat@gmail.com (I.D.V.); nagy.csaba@student.uoradea.ro (C.N.); 3 Faculty of Medicine and Pharmacy, University of Oradea, 1st December Square 10, 410028 Oradea, Romania; ganea.mada@yahoo.com (M.F.G.); ghitea.marc@gmail.com (M.C.G.); ghitea.evelinclaudia@gmail.com (E.C.G.); giteamaria28@gmail.com (M.F.G.); 4 Department of Pharmacy, Faculty of Medicine and Pharmacy, University of Oradea, 1st Decembrie Street, 410073 Oradea, Romania; corinamoisa@hotmail.com (C.M.); mganea@uoradea.ro (M.G.); 5 Preclinic Department, Faculty of Medicine and Pharmacy, University of Oradea, 1st Decembrie Street, 410073 Oradea, Romania; diana_pelea@uoradea.ro (D.C.P.); dobjanschil@yahoo.com (L.D.);

**Keywords:** oregano oil, salivary pH modulation, antimicrobial properties, oral microbiome, hormonal influences

## Abstract

**Background:** Salivary pH plays a critical role in oral health by influencing enamel demineralization, buffering capacity, and the ecology of oral microbiota. Essential oils such as *Origanum vulgare* (oregano) possess well-documented antimicrobial properties that may reduce acidogenic bacterial activity. However, the effects of edible delivery systems like jellies on salivary pH modulation and their potential interactions with hormonal states remain poorly understood. **Methods:** This study evaluated the in vitro antimicrobial activity of an oregano-oil-based jelly formulation against standard bacterial (*Staphylococcus aureus*, *Streptococcus pyogenes*, and *Escherichia coli*) and fungal (*Candida albicans*) strains using the Kirby–Bauer disc diffusion method. Additionally, a human trial (*n* = 91) measured salivary pH before and after administration of the oregano-oil jelly. Participants were characterized by age, smoking status, menopausal status, and presence of menstruation. Multiple linear regression was used to identify predictors of final salivary pH. **Results:** The oregano-oil jelly demonstrated strong in vitro antimicrobial activity, with inhibition zones up to 8 mm for *E. coli* and *C. albicans*. In vivo, mean unstimulated salivary pH increased from 6.94 to 7.07 overall, indicating a mild alkalinizing effect. However, menstruating participants showed a significant decrease in final pH (from 7.03 to 6.78). Multiple regression identified menstruation as a significant negative predictor (β = −0.377, *p* < 0.001) and initial pH as a positive predictor (β = +0.275, *p* = 0.002). Menopausal status was not a significant predictor, likely due to the small sample size. **Conclusions:** Oregano-oil jellies may represent a promising natural approach to support oral health by increasing salivary pH and providing strong antimicrobial activity. However, physiological states such as menstruation can significantly modulate this response, underscoring the importance of personalized or phase-aware oral care strategies. Further studies with larger, diverse cohorts and controlled hormonal assessments are needed to validate these findings and optimize product formulations.

## 1. Introduction

Salivary pH plays a key role in maintaining oral homeostasis by influencing microbial balance, dental demineralization, and remineralization processes, as well as overall cariogenic risk. Salivary acidification, primarily caused by the metabolism of acidogenic bacteria, is a major factor in the etiology of dental caries. Consequently, strategies aimed at controlling salivary pH by reducing acidogenic bacterial load are of significant interest in the prevention of oral diseases [1,2,3,4,5].

Oregano oil (*Origanum vulgare*) contains bioactive compounds such as carvacrol and thymol, which are known for their strong antimicrobial properties. Although there are no extensive clinical studies evaluating the specific effects of oregano-oil oral jellies on salivary pH, the existing literature suggests several relevant mechanisms. Research on extracts and essential oils with antibacterial activity in the oral cavity indicates a reduction in bacterial acidification through the inhibition of acidogenic species [6,7]. Additionally, studies on lozenges containing essential oils have reported moderate alkalinizing effects on salivary pH, attributable to reduced bacterial acid production. However, these effects are often transient and significantly influenced by the formulation characteristics of the product, including its composition (sugar content or non-fermentable sweeteners), intrinsic pH, and ability to stimulate salivary flow [8,9,10,11,12].

Hormonal influences on the oral environment are well recognized, particularly in relation to sex hormones, such as estrogen, progesterone, and testosterone [13]. Estrogen and progesterone receptors are present in salivary glands, and fluctuations in their levels—such as those occurring during the menstrual cycle, pregnancy, or menopause—can affect both the quantity and composition of saliva [14]. These changes may include altered salivary flow rate, decreased buffering capacity, and shifts in pH, all of which can influence microbial colonization and biofilm formation in the oral cavity. Estrogen deficiency, as seen in menopause, has been associated with reduced salivary output and an increased risk of oral dryness, caries, and periodontal disease. Meanwhile, menstrual-cycle-related changes have been linked to increased gingival inflammation and transient shifts in oral microbiota. Although less studied, testosterone may also play a modulatory role in oral immunity and salivary gland function, especially in males. These hormonal variations may, thus, impact the efficacy of antimicrobial interventions and should be considered when evaluating natural oral-care products [15].

In this context, investigating the impact of oregano-oil oral jelly on salivary pH is warranted and may contribute to the development of innovative strategies for oral disease prevention.

The aim of this study was to evaluate the effect of oregano-oil-based jelly formulations on salivary pH before and after oral administration, with a particular focus on how physiological variables such as menstruation and menopausal status may modulate this response. Additionally, in vitro assays were performed to assess the strong antimicrobial activity of the jelly formulation against reference bacterial and fungal strains. By integrating laboratory testing with in vivo salivary measurements in human participants, this study sought to provide initial evidence supporting the feasibility of using oregano-oil jellies as natural, functional oral-care products capable of modulating salivary pH and reducing microbial acidogenesis.

## 2. Materials and Methods

A prospective observational study was conducted over a period of 7 days at the University Dental Clinic. A total of 91 participants were recruited and provided informed consent. All participants were evaluated at baseline and after the intervention period for salivary pH measurements following the administration of the oregano-oil jelly.

Inclusion criteria included adults aged 18 years and older who were able to comply with study instructions and who had no significant systemic illness that could affect salivary secretion or oral health.

Exclusion criteria were the presence of active oral ulcerations, known allergies to oregano oil or any jelly ingredients, current use of systemic antibiotics or antifungal medication within the past month, and conditions associated with severe xerostomia or salivary gland dysfunction.

Participants were instructed to avoid food, beverages (other than water), and smoking for at least one hour prior to each saliva collection. All procedures were approved by the institutional ethics committee, and the study adhered to the principles of the Declaration of Helsinki.

### 2.1. Clinical Application of Oregano-Oil Jelly

In the clinical study, the oregano-oil jelly (P1) was used as the test product. The formulation consisted of *Origanum vulgare* essential oil from Sigma-Aldrich^®^ [St. Louis, MO, USA] (concentration: 40 µg per 6 mm disc equivalent), combined with gellan and pectin as gelation agents. Participants received daily single-use doses of approximately 5 g of jelly, instructed to dissolve it slowly in the oral cavity without chewing. Use was self-administered under standardized conditions, following instructions to avoid eating, drinking, or oral hygiene practices for at least 30 min before and after administration. The intervention period lasted 7 consecutive days.

### 2.2. Preparation and Composition of Oregano-Oil Jelly

The experimental jellies were formulated by combining *Origanum vulgare* essential oil with gellan and/or pectin to create a stable matrix with potential antibacterial and antifungal activity. The oregano-oil concentration used for laboratory testing was standardized at 40 µg/disc.

The following two test products were evaluated:

- P1: Jelly formulated with *Origani aetheroleum* (oregano essential oil);
- P2: A commercially available jelly product.

The commercially available jelly (P2) used for comparison was acquired from a national distributor [Sigma-Aldrich/Merck KGaA, Darmstadt, Germany], and its composition included a fruit-based pectin matrix with flavoring agents. According to the product label, it did not contain essential oils. No oregano oil was detected by the supplier in its composition. The pH of P1 was 5.6 and that of P2 was 5.2, measured prior to administration using a calibrated pH meter.

The intrinsic pH of the oregano-oil jelly (P1) was measured as 5.6, while the commercial jelly (P2) had a slightly lower pH of 5.2. These values were determined using a calibrated pH meter prior to administration. Despite their mildly acidic nature, the jellies did not lower salivary pH in most participants, suggesting that their effect on oral pH is not due to direct acid–base interactions.

### 2.3. Microbial Strains

The antimicrobial activity was tested against standard reference strains, including the following:

- Gram-positive bacteria: *Staphylococcus aureus* ATCC 25923 (LOT: 693518) and *Streptococcus pyogenes* ATCC 19615 (LOT: 701224);
- Gram-negative bacteria: *Escherichia coli* ATCC 35218 (LOT: 699211);
- Fungus: *Candida albicans* ATCC 14053 (LOT: 332-131-2).

### 2.4. Laboratory Testing Procedures

Antifungal activity was assessed on *Sabouraud agar* plates, while antibacterial activity was tested on Mueller-Hinton agar. The standardized disc diffusion method (Kirby–Bauer) was applied following the protocol described by Hudzicki (American Society for Microbiology, 2016) [16].

Bacterial and fungal suspensions were prepared in sterile 0.9% saline and adjusted to a 0.5 McFarland standard. Agar plates were inoculated with 20 µL of microbial suspension and allowed to solidify at room temperature for 15 min. Sterile 6 mm paper discs were impregnated with 40 µg of each test solution (P1 or P2) and placed on the agar surface.

Reference substances included:

- Clindamycin (2 µg/disc) and gentamicin (120 µg/disc) for bacteria;
- Nystatin (100 IU/disc) for *Candida albicans*.

### 2.5. Incubation and Assessment of Antimicrobial Activity

All plates were incubated at 37 °C in aerobic conditions for 24 h. *Streptococcus pyogenes* was incubated under 5% CO_2_-enriched conditions to ensure optimal growth, while *Candida albicans* was grown on *Sabouraud agar* under standard aerobic conditions. Antimicrobial activity was evaluated by measuring the diameter of the inhibition zones, including the 6 mm disc diameter.

The agar medium was poured to a uniform depth of approximately 4 mm, as recommended by CLSI guidelines, to ensure accurate diffusion and zone measurement. All in vitro tests were conducted in triplicate to ensure reproducibility. Each test plate contained both P1 and P2 discs placed equidistantly on the same agar surface to ensure identical growth conditions and facilitate comparative zone measurement.

### 2.6. Saliva Collection and pH Measurement

Unstimulated whole saliva was collected using the drooling technique into sterile containers. Participants were instructed to avoid food, beverages, and smoking for at least one hour prior to collection.

Salivary pH was measured using a calibrated digital pH meter, following recommended calibration procedures. All measurements were performed by a single trained operator to minimize variability.

Resting salivary flow rates and buffering capacity were not measured in this study. However, as these factors may significantly influence salivary pH and antimicrobial action, their potential confounding effects are acknowledged and discussed below.

Menstruation status was self-reported by female participants on the day of each saliva collection (T0 and T1). Only participants who reported active menstruation at the time of saliva collection were included in the “menstruating” subgroup analysis. Participants whose menstruation occurred between the two time points or on days other than saliva collection were not categorized as menstruating for the purpose of this study.

### 2.7. Statistical Analysis

Descriptive statistics were used to summarize participant characteristics and salivary pH values. Differences between groups (e.g., menopausal status and menstruation status) were evaluated using appropriate tests (e.g., *t*-tests or ANOVA, where applicable). Pearson correlation coefficients were calculated to assess associations among continuous variables. Multiple linear regression analysis was used to identify predictors of final salivary pH. All statistical analyses were performed using standard software packages (e.g., SPSS [version 20, New York, NY, USA], R, [version 4.3.2; R Foundation for Statistical Computing, Vienna, Austria] or Python [version 3.11]). A *p*-value < 0.05 was considered statistically significant.

No formal power calculation was conducted for this study, as it was designed as an exploratory investigation. The sample size of 91 participants was determined based on feasibility considerations, recruitment capacity, and reference to previous pilot studies evaluating salivary pH modulation interventions. This sample was considered sufficient to detect trends in pH variation and support hypothesis generation for future, more rigorously powered trials.

## 3. Results

### 3.1. Participant Characteristics

The study included 91 participants with a mean age of 24.5 ± 8.0 years (range: 19–57 years). The majority were female (76%) and lived in urban areas (86%). The proportion of smokers was 51%. Only 4 participants (4%) were postmenopausal, while 12 (13%) reported current menstruation at the time of testing (Table 1).

**Figure 1:**
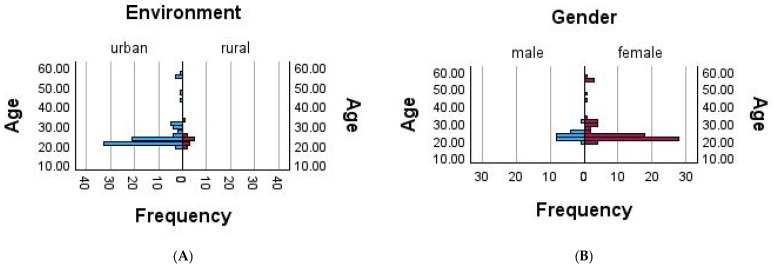
Age distribution of participants by (**A**) environment (urban/rural) and (**B**) gender (male/female).

The age distribution by environment (urban vs. rural) is presented in Figure 1A. The horizontal bar graph shows participant frequency across age groups, with the majority of younger participants residing in urban areas.

The age distribution by gender (male vs. female) is described in Figure 1B. The horizontal bar graph indicates a higher number of younger female participants, illustrating the sample’s demographic skew.

### 3.2. Salivary pH Before and After Oregano-Oil Jelly Use

The overall mean initial salivary pH was 6.94, increasing to 7.07 after administration of the oregano-oil jelly.

When stratified by menopausal status, participants without menopause showed an increase from 6.95 to 7.07, while postmenopausal participants had lower values overall and minimal change (6.75 to 6.68).

Menstruation status had a clear influence: those not menstruating increased from 6.93 to 7.10, while those menstruating showed a decrease from 7.03 to 6.78 (Table 2).

**Figure 2:**
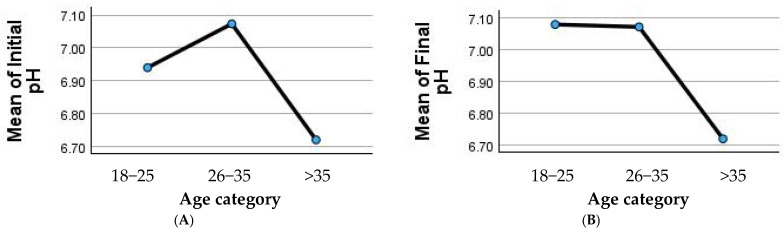
(**A**) Mean initial salivary pH and (**B**) mean final salivary pH after oregano-oil jelly administration, stratified by age category.

Panels (A) and (B) in Figure 2 illustrate the mean salivary pH values stratified by age category. In (A), the line graph shows the mean initial salivary pH across age groups (18–25, 26–35, and >35 years), with the highest pH observed in the 26–35 group, followed by a decline in participants over 35 years. Panel (B) presents the mean final salivary pH after oregano-oil jelly administration, indicating that pH remained relatively stable in younger participants (18–35 years) but dropped markedly in those over 35 years, suggesting age-related differences in salivary buffering response.

### 3.3. Correlations Among Variables

Pearson correlations showed that age was negatively correlated with final salivary pH (ρ = −0.25), while initial pH was positively correlated with final pH (ρ = +0.31). No significant correlation was observed between age and initial pH (Table 3).

### 3.4. Multiple Linear Regression

A multiple linear regression model was used to assess predictors of final salivary pH after oregano-oil jelly administration. The model explained 28% of the variance (R^2^ = 0.279, *p* < 0.001).

Menstruation at the time of testing was a significant negative predictor (β = −0.377, *p* < 0.001), indicating a substantial decrease in final pH. Initial salivary pH was a significant positive predictor (β = +0.275, *p* = 0.002). Age, smoking, and menopausal status were not significant predictors (Table 4).

**Figure 3:**
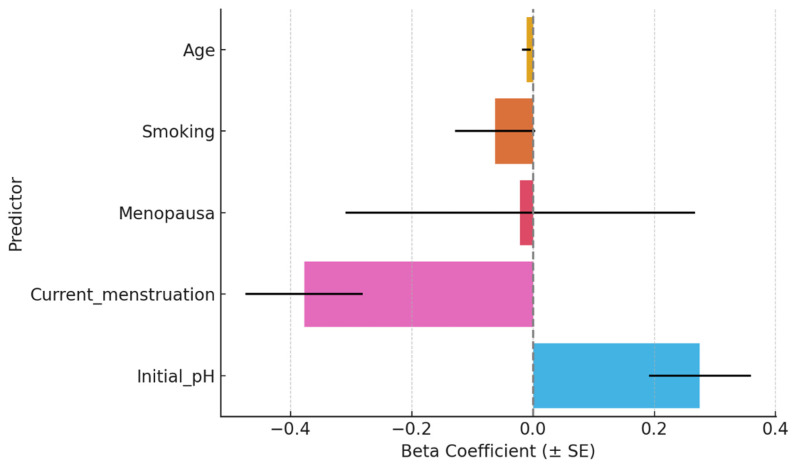
Regression coefficients for final salivary pH.

Menstruation was associated with a significant reduction in the salivary pH buffering response to oregano-oil jelly, while higher initial pH levels predicted greater post-intervention pH. Other factors, such as age, smoking, and menopause, showed no significant effects (Figure 3).

### 3.5. Sex-Based Differences in Salivary pH Response

**Figure 4:**
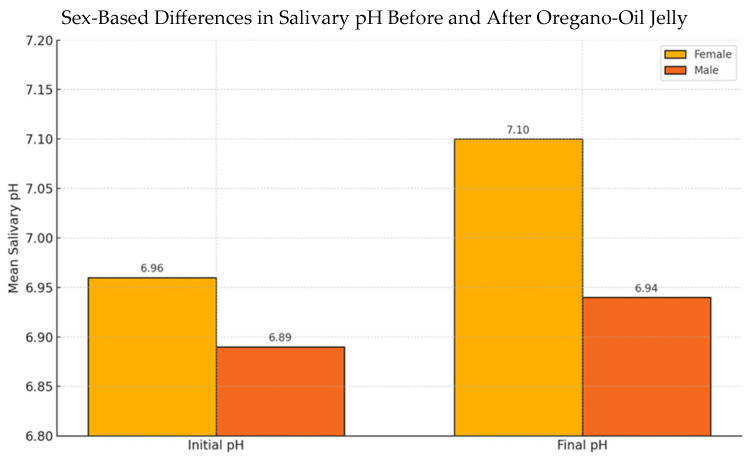
Mean salivary pH in male and female participants before and after oregano-oil jelly administration. Female participants showed a greater increase in pH (+0.14) compared to males (+0.05), though the difference was not statistically significant.

The influence of participant sex on salivary pH was evaluated (Figure 4). At baseline, mean unstimulated salivary pH was slightly higher in females (6.96 ± 0.21) compared to males (6.89 ± 0.18), but this difference was not statistically significant (*p* = 0.12). After oregano-oil jelly administration, females showed a mean increase of +0.14 pH units, while males exhibited a smaller mean increase of +0.05. The difference in response between sexes did not reach statistical significance (*p* = 0.08), though the trend suggests a potentially reduced buffering effect in males. No significant sex-related differences were observed in antimicrobial inhibition zone diameters.

### 3.6. Experimental Results of Antimicrobial Testing

P1 (oregano-oil jelly):

- *Staphylococcus aureus*: 34 mm;
- *Streptococcus pyogenes*: 35 mm;
- *Escherichia coli*: 8 mm;
- *Candida albicans*: 8 mm.

P2 (commercial jelly):

- *Staphylococcus aureus*: 22 mm;
- *Streptococcus pyogenes*: 25 mm;
- *Escherichia coli*: 22 mm;
- *Candida albicans*: 19 mm.

Reference antibiotics:

- Clindamycin: 27 mm (*S. aureus*), 26 mm (*S. pyogenes*);
- Gentamicin: 33 mm (*S. aureus*), 28 mm (*S. pyogenes*), 24 mm (*E. coli*);
- Nystatin: 20 mm (*C. albicans*).

Control discs containing only the gellan and pectin base matrix (without oregano oil) were also tested to rule out potential intrinsic antimicrobial effects of the gel base. No significant inhibition zones were observed for these control discs, confirming that the observed activity was attributable to the oregano-oil content.

## 4. Discussion

### 4.1. Main Findings

This study evaluated the effect of an oregano-oil jelly formulation on unstimulated salivary pH and found that, overall, administration increased mean pH from 6.94 to 7.07, consistent with a mild alkalinizing effect. While this shift may appear modest, even small increases in salivary pH can enhance buffering capacity and reduce the risk of enamel demineralization following sugar exposure. Saliva plays a central role in maintaining oral homeostasis, and a rise in resting pH may help shift conditions away from the critical demineralization threshold (typically pH 5.5–5.7), particularly in individuals exposed to frequent acid challenges. However, this pH response varied by physiological state: menstruating participants exhibited a paradoxical decrease in final pH (from 7.03 to 6.78), and postmenopausal participants showed minimal or negative change (6.75 to 6.68), suggesting hormonal modulation of oral buffering mechanisms.

Multiple linear regression confirmed menstruation as a significant negative predictor of final salivary pH, while higher initial pH values predicted a greater alkalinizing response. Age, smoking status, and menopausal status were not significant predictors in the multivariate model, likely reflecting sample distribution and power limitations.

### 4.2. Comparison with Previous Studies

Our observation of an overall increase in salivary pH after oregano-oil jelly administration is consistent with the documented antibacterial effects of *Origanum vulgare* essential oil. Prior in vitro studies have demonstrated strong antimicrobial activity of carvacrol and thymol, its primary active components, against Streptococcus mutans and other acidogenic oral bacteria [17,18,19,20,21]. By reducing the acid-producing bacterial load, essential oils can indirectly contribute to pH buffering in the oral environment [22].

Studies using essential-oil-based mouthrinses or lozenges (e.g., containing thymol or eucalyptol) have similarly reported transient increases in salivary pH or reductions in bacterial acidogenesis [22,23]. In 2023, a study was presented on the relationship between age, gender, BMI, diet, salivary pH, and periodontal pathogenic bacteria in children and adolescents [24]. However, direct clinical evidence on edible or chewable matrices such as jellies is scarce. Our findings support the hypothesis that a delivery system based on gellan/pectin matrices can facilitate the release of oregano oil while maintaining antibacterial potential.

Notably, the strong in vitro inhibition zones observed for our oregano-oil jelly (e.g., >30 mm for Gram-positive bacteria and 8 mm for *E. coli* and *Candida albicans*) corroborate its strong activity, even exceeding standard antibiotics in some cases. These antimicrobial data are in line with prior disc diffusion assays reported for oregano oil, highlighting its potential as a natural oral-care ingredient [8,25,26,27].

Although the oregano-oil jelly had an intrinsic pH of 5.6, and the commercial jelly measured 5.2, the overall effect observed in vivo was an increase in salivary pH. This suggests that the alkalinizing response was not due to the jelly’s baseline pH but rather to its antibacterial effects—likely via the reduction in acidogenic oral bacteria. Essential oils such as carvacrol and thymol, the primary bioactive components of oregano oil, are known to suppress acid production by inhibiting bacterial metabolism, which may indirectly shift salivary pH toward neutrality.

### 4.3. Influence of Menstruation and Menopause

A novel finding of this study is the significant impact of menstruation on the salivary pH response. While salivary pH generally increased post-intervention in participants not menstruating, those reporting menstruation exhibited a decrease in final pH. This may reflect hormonal fluctuations associated with the menstrual cycle, which have been shown to alter salivary composition, buffering capacity, and flow rate [28,29,30]. Previous research has demonstrated reductions in salivary pH and increases in bacterial colonization during the luteal and menstrual phases [31,32], potentially counteracting the antimicrobial action of oregano oil.

The minimal pH response observed in postmenopausal participants may similarly relate to hormonal factors. Estrogen deficiency has been associated with reduced salivary flow, changes in glandular composition, and diminished buffering capacity [33,34,35,36]. Although our sample of postmenopausal participants was small (*n* = 4), this finding aligns with literature describing increased oral susceptibility to caries and infections in postmenopausal women [37,38,39,40].

Although sex differences were not statistically significant in this study, the trend toward a reduced salivary pH response in male participants may suggest differing salivary composition or flow rate influenced by androgenic regulation. Testosterone has been implicated in modulating salivary gland output, and sex-specific variations in oral microbiota have been reported in the literature. Future studies should further explore these differences using hormonal assays and microbial sequencing to determine whether sex-based variation alters the effectiveness of oregano-oil interventions.

In addition to female hormonal influences, male sex hormones may also impact salivary physiology. Testosterone has been shown to affect salivary gland function, influencing both secretion rates and composition. Although males composed 24% of the sample, sex-based analyses revealed a smaller increase in salivary pH following oregano-oil jelly administration compared to females. While this trend did not reach statistical significance, it may reflect differences in salivary buffering or oral microbiota associated with androgenic influence. Future studies incorporating hormonal assays (e.g., serum or salivary testosterone, estrogen) are needed to elucidate these physiological interactions and potential differences in oral product efficacy by sex.

### 4.4. Strengths and Limitations

Strengths of this study include its integration of in vitro antimicrobial testing with in vivo salivary pH monitoring in a real-use scenario. The standardization of saliva collection and pH measurement protocols enhances internal validity.

However, limitations must be acknowledged. The sample was relatively young (mean age: ~24 years), with limited representation of postmenopausal individuals. Hormonal phase was self-reported, and menstrual cycle phase was not controlled beyond menstruation status. The study also did not include a placebo-controlled or blinded design. Future trials should include larger, more diverse samples and evaluate longer-term effects on oral microbiota and cariogenic risk.

Hormonal status (menstruating or postmenopausal) was based on self-reported data and not validated by hormonal profiling or menstrual phase tracking. This introduces a potential source of misclassification bias, especially in perimenopausal women or those with irregular cycles. Furthermore, the number of postmenopausal participants was very low (*n* = 4), limiting the ability to draw robust conclusions regarding menopausal effects on salivary pH or product response. Future studies should employ objective hormonal measurements and ensure more balanced recruitment across age and reproductive status groups.

### 4.5. Implications and Future Research

These findings suggest oregano-oil jellies may offer a natural strategy to support salivary pH buffering by reducing acidogenic bacteria. However, physiological states such as menstruation may modulate this response, indicating the need for personalized or phase-aware oral care interventions. Future research should explore hormonal influences in greater detail, investigate microbiome changes, and test optimized sugar-free formulations to minimize potential acidogenic substrates.

It is important to clarify that while this study focused on unstimulated salivary pH, the Stephan curve—commonly referenced in discussions of dental caries risk—describes pH dynamics at the plaque–enamel interface, not in bulk saliva. Plaque pH can drop rapidly following carbohydrate exposure and remains the most direct indicator of demineralization risk. In contrast, salivary pH provides an indirect measure of the oral environment’s buffering potential. Although changes in salivary pH can suggest shifts in microbial acidogenicity or buffering capacity, they do not fully capture the localized effects occurring at the tooth surface. Future studies should include plaque pH measurements or microelectrode-based profiling to complement salivary data and more accurately assess the cariogenic potential of oral interventions.

## 5. Conclusions

This study demonstrates that oregano-oil jellies can exert a mild alkalinizing effect on salivary pH, supporting their potential role in modulating the oral environment and reducing acidogenic bacterial activity. Our findings highlight that salivary pH response is not uniform across physiological states: menstruating participants exhibited a significant reduction in final pH, suggesting hormonal modulation of buffering capacity and antimicrobial efficacy.

In vitro antimicrobial testing confirmed the strong, activity of the oregano-oil jelly formulation against Gram-positive and Gram-negative bacteria, as well as *Candida albicans*, exceeding or matching standard antimicrobials in some cases. These results support the feasibility of developing functional oral health products based on oregano essential oil incorporated into edible matrices.

However, variation in salivary pH response underscores the need for personalized approaches, especially considering hormonal status. Future research should involve larger, more diverse samples, control for menstrual cycle phases and menopausal status, and explore longer-term effects on oral microbiota and dental health outcomes. Optimizing sugar-free formulations and evaluating patient acceptability and safety will be essential steps toward clinical application. Future studies should also evaluate the safety profile of oregano-based formulations, especially regarding their use during pregnancy, in pediatric populations, in individuals with diabetes due to potential hypoglycemic effects, and in patients taking anticoagulant medications.

Overall, oregano-oil jellies represent a promising, natural, and potentially effective strategy for supporting oral health through targeted modulation of the oral microbiome and pH balance.

## Figures and Tables

**Table 1:** Demographic characteristics of participants.

Variable	*n* (%) or Mean ± SD
Age (years)	24.5 ± 8.0
Female	69 (76%)
Male	22 (24%)
Urban residence	78 (86%)
Rural residence	13 (14%)
Smokers	46 (51%)
Non-smokers	45 (49%)
Postmenopausal	4 (4%)
Current menstruation	12 (13%)

**Table 2:** Salivary pH (mean values) before and after oregano-oil jelly stratified by menopausal and menstruation status.

Group	Initial pH
Total sample	6.94
No menopause	6.95
Menopause	6.75
No menstruation	6.93
Current menstruation	7.03

**Table 3:** Pearson correlation coefficients.

Variables	Correlation (ρ)
Age vs. Final pH	−0.25
Initial pH vs. Final pH	+0.31
Age vs. Initial pH	−0.08

**Table 4:** Multiple linear regression results predicting final salivary pH.

Predictor	β Coefficient	*p*-Value
Age	−0.011	0.143
Smoking	−0.062	0.351
Menopausal status	−0.021	0.942
Current menstruation	−0.377	<0.001 *
Initial pH	+0.275	0.002 *

* Significant at *p* < 0.05.

## Data Availability

All the data processed in this article are part of the research for a doctoral thesis, which is being archived in the medical nutrition office where the interventions were performed.
